# Posterior Vertebral  Column  Resection for Severe Spinal Deformity Correction: Comparison of Pediatric, Adolescent, and Adult Groups

**DOI:** 10.1155/2022/5730856

**Published:** 2022-09-21

**Authors:** Zhibo Song, Zhaoquan Zhang, Xiaochen Yang, Zhi Zhao, Tao Li, Ni Bi, Jingming Xie, Yingsong Wang

**Affiliations:** Department of Orthopaedics, The Second Affiliated Hospital of Kunming Medical University, Kunming, China

## Abstract

We compared the pre-, intra-, and postoperative characteristics among three groups of patients who underwent posterior vertebral column resection (PVCR) to clarify age-related characteristics and to guide patient management, surgical planning, and complication avoiding. We compared and analyzed the etiology, surgical events, outcomes, and complications among pediatric, adolescent, and adult patients who underwent PVCR in a single-center database retrospectively. Patients were categorized into pediatric (0–12 yr), adolescent (13–19 yr), and adult (>20 yr) cohorts. Demographics, surgical events, clinical and radiographic results, and major complications were compared between groups. A total of 87 patients with a mean follow-up 42 (24–96) months were identified. Pediatric group (14) had a high frequency of congenital vertebral and cardiac abnormal, adolescents (47) presented more intracanal malformations, and idiopathic was common in the adult group (26). Although pediatric patients had shorter fusion levels than adolescent and adult, their mean resected vertebrae (1.91), percentage of blood loss (estimated blood loss per total blood volume) (201.9%), and operative time were much higher. The coronal/sagittal correction rate was significantly higher in the pediatric group (73.6%/72.3%). Overall, surgical complications were more frequent in adults, particularly neuromonitoring alert and implant failure. However, more severe complications were noted in younger patients. For pediatric patients with PVCR, poor physiological conditions and frequent comorbidities indicated cautious patient selection and sufficient preoperative preparation. The higher correction rate may be due to the excellent compliance of the spinal cord. For adult patients, preoperative traction and adjusting the tension of the spinal cord during surgery could contribute to neurological safety.

## 1. Introduction

Posterior vertebral column resection (PVCR) has been extensively recognized as a powerful and aggressive technique for achieving optimal correction of severe and rigid spinal deformity [1–3]. Nevertheless, surgeons always remain cautious inpatient selection and surgical planning because of the high complication rate of PVCR. The operation divides the spine into two free segments (cephalic and caudal) connected only by the spinal cord, which might be stretched or displaced. Except for neurological risks, complex and lengthy procedures are another factor that may develop nonneuro complications. As reported, the total complication rate of PVCR ranges from 40% to 63%, it portends that more efforts are needed for reducing the complication rate [2–6].

Generally, it is believed that patients' risks and benefits were determined by etiology distribution, potential growth, and comorbidities situation, which may well vary with age-related differences [7, 8]. Series SRS data showed this differential in complications between adolescent, pediatric, and adult scoliosis patients. For adolescent, the overall complication rate reported by Coe et al. [9] was 5.7% of 6334 patients. Of 19360 children, 1,971 total complications (10.2%) occurred. As for adult, complications were present in 521 patients (10.5%), and a total of 669 complications were developed (13.4%) in 4980 cases [9–11]. Although risk factors such as preexisting neurologic dysfunction, massive blood loss, and poor cardiopulmonary function have been identified, the age-related differences in PVCR, which may affect the preparation and outcomes, were little described and compared [12].

The purposes of this study were to compare the clinical outcomes and related complications of PVCR of severe and rigid deformities between pediatric, adolescent, and adult patients and search for the age-related differences. We hypothesized that PVCR was an effective means with good radiological outcomes and high complication rate in each age group.

## 2. Materials and Methods

PVCR is defined as a complete circumferential resection of one or more vertebra with all elements and adjacent discs through posterior approaches only. The final surgical indication was based on rigid deformity conditions, neurological function, pulmonary function, and life expectancy, which other osteotomies could not improve after adequacy evaluation(serial casting and bracing, traction, respiratory training). In detail, we included patients as follows: severe and rigid spinal deformity, the main curve greater than 100° with restricted flexibility; large curve (more than 80°) with rapid progression, syringomyelia, Chiari malformation, tethered cord (with increased spinal cord tension), ongoing neurological deficits, or progressive lung volume decrease. Patients who underwent posterior hemivertebra resection, or had a history of anterior or posterior spinal surgery, degenerated pathogenesis, or performed by other osteotomies were excluded from this study.

All operations were performed at a single institution from 2004 to 2016. Before data collection and analysis, we obtained approval from the institutional review board. The assessment includes etiology, radiography, neurology, nutriology, and viscera reserve function. We used standing anterior-posterior and lateral spinal radiographs, and the main curve was measured by the Cobb method and identified as kyphoscoliosis (coronal and sagittal planes curves) or kyphosis only. Moreover, the coronal and sagittal balance was assessed by the distance of the C7PL offset CSVL line and SVA. Noninvasive bilevel positive airway pressure ventilation or lip contraction breathing were carried out routinely for perioperative pulmonary function exercise. All malnourished patients were either receiving enteral nutrition via nasointestinal tube or parenteral nutrition during the preparation period. Preoperative traction was implemented for nearly all the cases. It started from a small weight (3 kg on the skull and 6 kg on the femur) and gradually reached a maximum of 50% of body weight as the reflection of traction, which means no progressed neuro deficits and normal physiological functions (respiration, circulation, digestion, and metabolism). When the process lasted 1–2 weeks at the maximum traction weight, we organized multidisciplinary consultation (cardiology, neurology, intensive care, respiratory, and anesthesiology) to evaluate surgical risks and to determine whether to perform surgery or not.

The procedure was performed as previously reported [13]. Briefly, an incision was made through the shape of scoliosis, and the lateral edges of the lamina were exposed from connective tissue. Next, higher density screws were inserted in vertebrae as much as possible by the free-hand technique. The temporary fixation rod should be placed before the vertebral column resection is completed to ensure the stability of the spine. After the posterior column structure is resected, the anterior and middle column structures of the spine are exposed and removed following removing the transverse process and ribs on the convex and then the concave side. We moderately shorten the spine by compressing the space created by VCR to reduce the tension and increase the compliance of the spinal cord before correction. Techniques of in-situ rod bending, alternative rod changing, distraction, compression, closing, and opening are used for correction. Particularly, we used the percentage of blood loss (estimate blood loss/total blood loss) to evaluate to avoid confounding bias.

Patients were categorized into pediatric, aged 0–12 years, adolescent, aged 13–19 years, and adult, older than 20 years, cohorts. Demographic data and deformity status were reviewed in all patients. Surgical characteristics, including resected vertebrae, fixed levels, estimated blood loss per total blood volume, and operation duration, intraoperative neuromonitoring (IONM) including somatosensory evoked potential (SSEP) and transcranial motor evoked potentials (TcMEP) changes, were collected. Postoperatively, the prevalence of transit and permanent complications and changes in pulmonary function parameters were reviewed during follow-up.

Statistical analysis was performed using the statistical package for the Social Sciences, v13.0 (IBM Corp., Armonk, NY, USA). Data were analyzed using the Fisher exact test for dichotomous variables, and independent *t*-tests or Wilcoxon signed-rank test was used to compare groups. Statistical significance was defined as *P*-value of less than 0.05.

## 3. Results

As shown in Table 1, a total of 87 patients who underwent PVCR between October 2004 and January 2016, with mean age 18.7 years (range, 9 to 45 years) and mean follow-up of 42 months (range, 24 to 96 months), were identified. Etiologic diagnoses were nonidiopathic (57 patients, 65.5%) including congenital deformity, sequela of spinal *tuberculosis*, neuromuscular, neurofibromatosis, associated with Chiari malformation and/or syringomyelia, ankylosing spondylitis, and idiopathic deformities (30 patients, 34.5%). The distribution of the curve shape was similar in both three groups, and kyphoscoliosis type A made the maximum proportion. Preoperatively, the most common medical association was an intracanal anomaly (23 patients, 26.4%) detected by magnetic resonance imaging. Echocardiography indicated cardiac structure or functional parameters abnormality, regardless of the history of heart operation, in 11 patients (12.6%). Pulmonary function tests showed restrictive respiratory impairment in a large proportion of patient, with average predicted forced vital capacity (FVC%) as 51.3 ± 14.7% and forced expiratory volume in 1 second (FEV 1.0%) as 52.6 ± 16.2%. A number of 14 patients were characterized by severe ventilatory dysfunction, and three of them manifested with significant hypoxemia.

Surgical characteristics showed the mean resected vertebral was 1.34, and the extent of fusion was 13.3 vertebrae (range 7–17 vertebrae). The mean operating time was 595 minutes (range 320–920 minutes), and the mean operative estimated blood loss calculated as the ratio of estimated blood loss to estimated body blood volume was 158.8% (range 18%–570%). The mean major curve deformity of scoliosis was 108.7° ± 24.5° preoperatively and decreased to 36.2° ± 12.4° at the final follow-up, with a mean correction rate of 66.7%. The segmental kyphosis corrected from 89.7° ± 28.1° preoperatively to 29.8° ± 14.1° at the final follow-up, with a correction rate of 66.8%.

There was no permanent neurologic deficit encountered in all patients. Transient intercostal neuralgia (4 patients, 4.6%) and transient spinal cord dysfunction noticed by wake-up abnormal (2 patients, 2.3%) and postoperative ischemia-reperfusion injury (1 patient, 1.1%) were returned to normal within no longer than three months after the operation. During the procedure, SSEP warning (3 patients, 3.4%) and SSEP/TcMEP warning (8 patients, 9.2%) were detected, in patients who could collect monitoring data since 2011.

As a whole, there were a total of 20 patients have been recorded with 25 major nonneurological complications, for an overall incidence of 23.0%, including respiratory complications (11 patients, 12.6%), cardiovascular adverse events or irreformable hypotension (6 patients, 6.9%), malignant hyperthermia (1 patient, 1.1%), wound infection (3 patients, 3.4%), and optic deficit (1 patient, 1.1%).

Pedicle screw-related pedicle wall perforation rate was similar in three groups. During the follow-up period, three patients have been recorded as implant failure, including one-rod fracture at PVCR site (in situ bending rod and not exchanged), one-rod fracture at caudal fixation segment, and one ultimate cephalic screw pull-out following deep infection. All implant failures occurred within the first year postoperative, and no more implant complication after revision surgery. Moreover, at the most recent follow-up, PFT parameters indicated average FVC% and FEV 1.0% were both improved than preoperative in three groups (Table 1).

### 3.1. Pediatric Group

Overall, pediatric patients who underwent PVCR had the highest frequency of congenital vertebrae (7/14, 50.0%) and sequela of spinal *tuberculosis* (3/14, 21.4%) in nonidiopathic subgroup and compared to other groups. There was no one patient diagnosed as idiopathic scoliosis. Comorbidity with cardiac abnormalities (4/14, 28.6%) was much higher in this group.

Although pediatric patients had shorter fusion levels than adolescent and adult patients (11.8 vs. 14.3, 13.4), the mean resected vertebrae (1.91), estimated blood loss volume (201.9%), and operative time (644 min) were much higher. The coronal and sagittal correction rate (73.6% and 72.3%) was significantly higher in the pediatric group.

**Figure 1:**
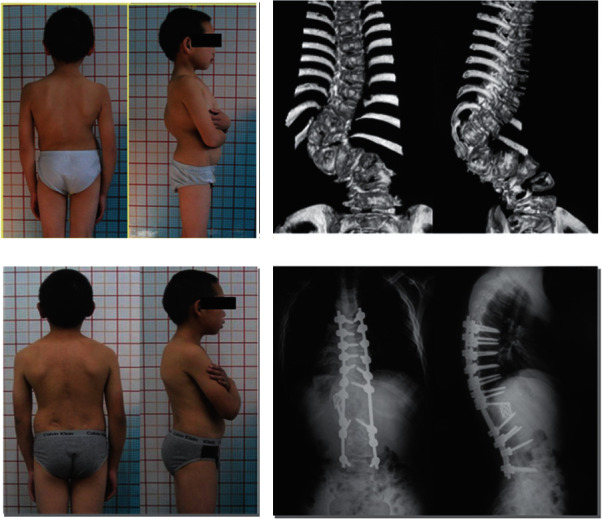
The patient was a 9 years old boy with lumbar congenital scoliosis. (a) Clinical feature preoperative. (b) Computer tomographic with 3-dimensional reconstruction showed multiple complex congenital deformity. (c) Clinical feature at 2 years follow-up after PVCR. (d) X-ray of anterior-posterior and lateral at 2 years follow-up.

Only one pediatric patient (1/14, 7.1%) occurred IOM alert, and 2 with nonneurological complications (2/14, 14.3%). At the most recent follow-up, pediatric patients showed the significant improvement in pulmonary function than other groups. However, more severe medical complications, such as malignant arrhythmia and malignant hyperthermia, were noted in younger patients (Figure 1).

### 3.2. Adolescent Group

Overall, adolescent patients who underwent PVCR had the largest percentage of all patients (47/87, 54.0%), thus either adolescent idiopathic (18/87, 20.7%) or nonidiopathic patients (31/87, 35.6%) occupied a large proportion in all patients. Comorbidity with intracanal anomaly (14/47, 29.8%) was much higher in this group.

**Figure 2:**
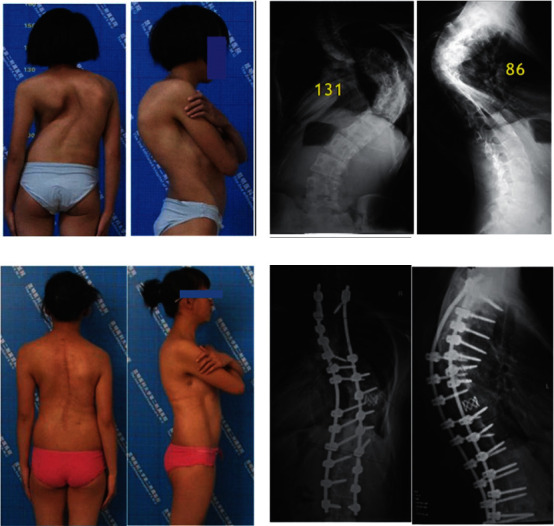
A 17-year-old girl with thoracic kyphoscoliosis. (a) Clinical feature preoperative. (b) X-ray of anterior-posterior and lateral preoperative. (c) Clinical feature at 2 years follow-up after PVCR. (d) X-ray at 2 years follow-up.

Nonneurological complications encountered in 8 adolescent patients (8/47, 17.0%), and 3 IONM alert (3/47, 6.4%). No implant-related complication during follow-up was recorded (Figure 2).

### 3.3. Adult Group

**Figure 3:**
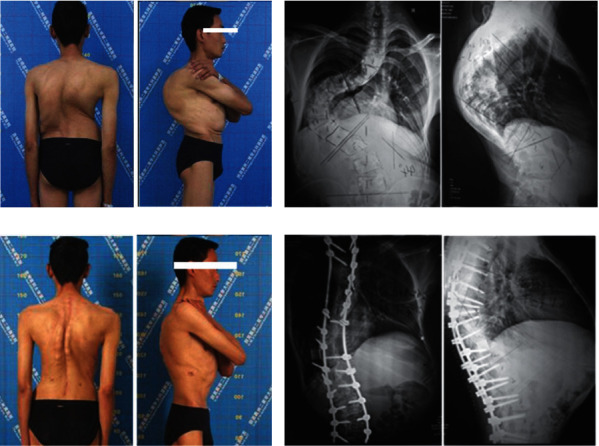
The patient was 35-year-old adult with severe and rigid deformity. (a) Clinical feature preoperative. (b) X-ray of anterior-posterior and lateral preoperative. (c) Clinical feature at 2 years follow-up after PVCR. (d) X-ray at 2 years follow-up.

Overall, adult patients who underwent PVCR had the largest degree of preoperative curvatures. Surgical complications were more frequent in adult patients, particularly included IONM alert (7/26, 26.9%) and implant failure (3/26, 11.5%). There was the largest possibility of nonneurological complications in adult patients than in other group (Figure 3).

## 4. Discussion

For the patient with severe, rigid, and angular spinal deformity, the inherent advantage of PVCR induced effective correction, when the PVCR procedure is performed by an experienced surgical team. According to our experience for more than 10 years, after vertebral resection for creating a correction space, the torsional and abrupt spinal column is divided into two free segments on the cephalic and caudal sides and only connected by the spinal cord. Realignment of the spinal column is controlled by pedicle instrumentation, in order to keep the spinal temporary stabilization and preserve the corrective space not to closing. Compression for spinal shortening is helpful to reduce the tension of the spinal cord. However, closing the space may lead to excessive spinal cord translation and impingement. In fact, the key point of correction rate is the safety of the spinal cord, which depends on the maximum displacement that the spinal cord can tolerate. In the present study, the overall result of 87 patients showed more than 65% corrective rate on both coronal and sagittal planes (72° on coronal and 60° on sagittal), with a satisfactory global balance. Similarly, Yang et al. [14] recently systematically reviewed the articles about PVCR in the treatment of spinal deformity and calculated that correction of scoliosis was 64.1°, and correction of kyphosis was 58.9° in severe spinal deformity, accounting for a correction rate of 61.2 and 63.1%.

The requisite residual space, while the corrective process completes, determined the fundamental difference from PVCR to traditional three-column osteotomy (pedicle subtraction osteotomy, PSO). Kim et al. [15] reported a 30°–40° correction effect by PSO in a retrospective review of 140 patients. A comparison of the radiological outcomes between PSO and VCR from a single institution also indicated that VCR facilitated significantly greater correction of segmental kyphosis at the level of the osteotomy than PSO (VCR: 64.0° vs. PSO: 35.2°) [16]. In addition, closing-opening wedge osteotomy has been reported extended correction rate for kyphotic curves [17, 18]. After the first 30°–35° correction by closing, a titanium cage insertion moved the hinge to the spinal cord, and the opening wedge technique was used. However, prior support on the anterior column may not only restrict the adjustability of two segments of the spinal column and limited the further corrective dimension but also denied the maneuver to timely adjustment tension of a spinal cord. Multilevel osteotomies tend to benefit global curvature such as neuromuscular scoliosis [19]. The maximum proportion in our patients indicated that PVCR was applied to severe and rigid deformity, especially for kyphoscoliosis patients with more significant scoliotic curve, or those with sharp angular deformity.

Our result suggested that the coronal and sagittal correction rate was significantly higher in the pediatric group (73.6% and 72.3%) than adolescent and adult patients. The higher correction rate in pediatrics may be due to the residual elasticity of the spine and compliance of spinal cord. Research concerning the PVCR correction mechanism analyzed the changes of the Cobb angle in the major curve and each segment, including the upper, middle, and lower segments of the major curve. Although the middle segment offered the highest contribution rate (near 50%) to the deformity correction of the major curve, and correction from the middle segment depended on configuration change of correction space, correction on upper and lower segments of the major curve also made contribution [13]. Additionally, we prefer to choose bigger diameter screws and high screw density in pediatric PVCR patients, for reliable anchor placement [20].

We emphasized that our patients in the present study did not have simple congenital hemivertebra with the mild or moderate curve. Though the procedure of posterior hemivertebra resection was similar to PVCR in exposing and removing the deformed vertebra, the correction after hemivertebra resection totally differs from our PVCR for rigid and severe spinal deformity in that (1) the hemivertebra correction involves a wedge closing technique only, (2) the segments involved in correction and fixation are shorter, thus requiring much less operation time and blood loss, and (3) the neurological safety profile is obviously better [21]. Therefore, the pediatric group in the present study was only included severe and progression deformities involving long segments, which prompted surgeons to perform a positive intervention, in order to avoid worse graduation and extremely severe curves. Besides, for a child younger than 10 years, chosen vertebral column resection and long fusion are needed cautiously evaluate, in the risk of insufficient thoracic development. As a well-designed strategy, VCR following growth-friendly instrumentation in the younger patient was reported to preserve spinal growth [22]. Jeszenszky et al. [23] performed PVCR in early-onset spinal deformities and advocated that additional surgical procedures are often necessary during growth, and hence, nonfusion instrumentation beyond the vertebral resection site is advantageous, as it permits spinal growth and the later addition of fusion.

Our previous study revealed that although PVCR patients with severe rigid spinal deformity had had a significant decrease in PFTs at 2-week postoperation, they should be increased up to preoperative baseline at 1-year postoperative and significantly improved at 2 years after surgery. Pediatric patients who underwent PVCR showed the significant improvement in pulmonary function than other groups. The study by Bumpass et al. [8] reported that, in pediatric patients, PVCR resulted in small but significant improvements in postoperative PFTs. In adult patients, no significant increases in PFTs were found. Patients who have the greatest potential for lung and thoracic cage growth after spinal correction are most likely to have improved pulmonary function.

PVCR is a technically challenging procedure, and complications are common. There was no one permanent neurological complication in our series. However, our result indicated that IONM alert and transient neurological deficits were more frequent in adult patients. Adult spinal deformity patients underwent PVCR were noticed to associate with more intraspinal anomalies than the pediatric group. According to our previous study, PVCR related neurologic deficits risk factors included angular and abrupt curves, combined intraspinal anomalies with increased spinal cord tension, and preoperatively symptoms and signs of neurological dysfunction [24]. Moreover, the prolonged and gradually progressed spinal deformity may aggravate the distraction on the spinal cord. Commonly, around the apex region of scoliosis patients, the spinal cord was consistently located on the concave side in the spinal canal, which would minimize the spinal cord tension. The compensated ability was weakened with age growing.

Patients who underwent PVCR experienced an expected high rate of major nonneurological complications [2]. Our result revealed more respiratory and cardiovascular adverse events perioperative in adult patients. However, more severe medical complications were noted in younger patients, such as malignant arrhythmia and malignant hyperthermia. We attributed it to higher cardiac comorbidity and larger estimated blood loss volume (201.9%) in pediatric patients in our series.

In summary, poor physiological conditions and frequent comorbidities indicated cautious patient selection and sufficient preoperative preparation. Although the correction rate in the pediatric group was significantly higher, we still recommend to avoid the use of PVCR in children as the unpredictable serious complications and massive bring a high risk for surgery. For adult patients, preoperative translation and adjusting the tension of the spinal cord during surgery could contribute to neurological safety.

The main limitation of this study was the retrospective nature and historical control groups which may lead to a potential for uncontrolled selection bias and the small number of pediatric patients. The treatment of early-onset scoliosis is different from adolescent or adult scoliosis, and allowing the spine and lung development were more important than correcting the deformity. So only the severe and complex cases should have to undergo PVCR. Besides, children's spine is more flexible, and a satisfactory deformity correction could be obtained by SPO or PSO osteotomy. These are the factors for the small number of pediatric patients.

## 5. Conclusion

PVCR is an effective opinion with good radiological outcomes and high complication rate in both age groups. For pediatric patients, poor physiological status and major comorbidities indicated cautious patient selection for PVCR, and actively preoperative preparation. The higher correction rate in pediatrics may be due to the residual elasticity of the spine, and compliance of the spinal cord. For adult patients, preoperative traction and surgically adjusting the tension of the spinal cord have contributed to the neurological safety.

## Figures and Tables

**Table 1:** Comparison between patients in different age groups.

	Total (%)	Pediatric 0–12 yr(%)	Adolescent 13–19 yr(%)	Adult >20 yr(%)
Number *(n)*	87	14	47	26
Age *(yr)*	18.9	10.9	15.6	29.1

Etiology *(n)*				
*Nonidiopathic*	57*(65.5)*	14*(100* ^*∗*^)	31*(66.0)*	12*(46.2)*
*Congenital*	35	7	21	7
*TB*	13	3	5	5
*Idiopathic*	30*(34.5)*	0*(0.0)*	16*(34.0)*	14*(53.8∗*)

Curve categories *(n)*				
*Kyphosis*	14*(16.1)*	3*(21.4)*	5*(10.6)*	6*(23.1)*
*Kyphoscoliosis-Scoliosis larger*	48*(55.2)*	7*(50.0)*	24*(51.1)*	14*(53.8)*
*Kyphoscoliosis-Kyphosis larger*	25*(28.7)*	4*(28.6)*	18*(38.3)*	6*(23.1)*

Comorbidity *(n)*				
*Cardiac*	11*(12.6)*	4*(28.6∗*)	5*(10.6)*	2*(7.7)*
*Intracanal*	23*(26.4)*	2*(14.3∗*)	14*(29.8)*	7*(26.9)*
Resected seg *(n)*	1.34	1.91*∗*	1.21	1.29
Fixed segment *(n)*	13.3	11.8	14.3	13.4
EBL (per total blood volume %)	158.8	201.9*∗*	155.5	141.6
OP time *(Min)*	594.741	644*∗*	595.625	575.75
Precor cobb	108.7 ± 24.5	102.2 ± 29.3	109.0 ± 25.7	111.6 ± 23.3
Post-cor cobb	36.2 ± 12.4	27.0 ± 14.5	37.2 ± 15.3	39.3 ± 12.8
Corr. Rate %	66.7	73.6*∗*	65.8	64.9
Pre-sag cobb	89.7 ± 28.1	73.2 ± 21.8	92.6 ± 35.2	93.4 ± 24.9
Post-sag cobb	29.8 ± 14.1	20.3 ± 13.0	32.0 ± 14.7	30.8 ± 11.9
Corr. Rate %	66.8	72.3*∗*	65.4	67.0
Major nonneuro complication *(n)*	20*(23.0)*	2*(14.3)*	8*(17.0)*	10*(38.5∗*)
IOM abnormal *(n)*	11*(12.6)*	1*(7.1)*	3*(6.4)*	7*(26.9∗*)
Implant failure *(n)*	3*(3.4)*	0*(0.0)*	0*(0.0)*	3*(11.5∗*)
FVC% improve (%)	6.0	7.8	6.7	3.8*∗*
FEV% improve (%)	6.1	7.7	6.8	4.0*∗*

Note.  ^*∗*^ indicates significant statistical difference with *P* < 0.05.

## Data Availability

The dataset can be obtained from the corresponding author upon request.

## References

[1] Sucato D. J. (2010). Management of severe spinal deformity: scoliosis and kyphosis. *Spine (Phila Pa 1976)*.

[2] Wang Y., Xie J., Zhao Z. (2015). Perioperative major non-neurological complications in 105 patients undergoing posterior vertebral column resection procedures for severe rigid deformities. *Spine (Phila Pa 1976)*.

[3] Xie J., Wang Y., Zhao Z. (2012). Posterior vertebral column resection for correction of rigid spinal deformity curves greater than 100°. *Journal of Neurosurgery: Spine*.

[4] Lenke L. G., OʼLeary P. T., Bridwell K. H., Sides B. A., Blanke L. A., Blanke K. M. (2009). Posterior vertebral column resection for severe pediatric deformity: minimum two-year follow-up of thirty-five consecutive patients. *Spine 1976*.

[5] Lenke L. G., Newton P. O., Sucato D. J. (2013). Complications after 147 consecutive vertebral column resections for severe pediatric spinal deformity a multicenter analysis. *Spine 1976*.

[6] Kim S. S., Cho B. C., Kim J. H. (2012). Complications of posterior vertebral resection for spinal deformity. *Asian Spine Journal*.

[7] Diab M. G., Vitale J. M., Vitale M. G. (2011). The role of posterior spinal osteotomies in pediatric spinal deformity surgery. *Journal of Pediatric Orthopaedics*.

[8] Bumpass D. B., Lenke L. G., Bridwell K. H. (2014). Pulmonary function improvement after vertebral column resection for severe spinal deformity. *Spine 1976*.

[9] Coe J. D., Arlet V., Donaldson W. (2006). Complications in spinal fusion for adolescent idiopathic scoliosis in the new millennium a report of the Scoliosis Research Society Morbidity and Mortality Committee. *Spine 1976*.

[10] Reames D. L., Smith J. S., Fu K. M. G. (2011). Complications in the surgical treatment of 19,360 cases of pediatric scoliosis: a review of the Scoliosis Research Society Morbidity and Mortality database. *Spine*.

[11] Sansur C. A., Smith J. S., Coe J. D. (2011). Scoliosis research society morbidity and mortality of adult scoliosis surgery. *Spine*.

[12] Zhang Y., Xie J., Wang Y., Bi N., Li Z., Li T. (2014). Thoracic pedicle classification determined by inner cortical width of pedicles on computed tomography images: its clinical significance for posterior vertebral column resection to treat rigid and severe spinal deformities-a retrospective review of cases. *BMC Musculoskeletal Disorders*.

[13] Xie J., Li T., Wang Y., Zhao Z., Bi Y., Bi N. (2012). Change in Cobb angle of each segment of the major curve after posterior vertebral column resection (PVCR): a preliminary discussion of correction mechanisms of PVCR. *European Spine Journal*.

[14] Yang C., Zheng Z., Liu H., Wang J., Cho Y. J., Cho S. (2016). Posterior vertebral column resection in spinal deformity: a systematic review. *European Spine Journal*.

[15] Kim K. T., Lee S. H., Suk K. S., Jeong J. H., Jeong B. O. (2012). Outcome of pedicle subtraction osteotomies for fixed sagittal imbalance of multiple etiologies: a retrospective review of 140 patients. *Spine*.

[16] Auerbach J. D., Lenke L. G., Bridwell K. H. (2012). Major complications and comparison between 3-column osteotomy techniques in 105 consecutive spinal deformity procedures. *Spine*.

[17] Kawahara N., Tomita K., Baba H., Kobayashi T., Murakami T., Murakami H. (2001). Closing-opening wedge osteotomy to correct angular kyphotic deformity by a single posterior approach. *Spine*.

[18] Liu X., Yuan S., Tian Y., Wang L., Li Y., Li J. (2015). Expanded eggshell procedure combined with closing-opening technique (a modified vertebral column resection) for the treatment of thoracic and thoracolumbar angular kyphosis. *Journal of Neurosurgery: Spine*.

[19] Suh S. W., Modi H. N., Yang J., Jang H. R., Jang K. M. (2009). Posterior multilevel vertebral osteotomy for correction of severe and rigid neuromuscular scoliosis: a preliminary study. *Spine*.

[20] Xie J., Wang Y., Zhang Z., Zhang Y. (2011). The safe placement of upper and middle thoracic pedicle screws in pediatric deformity. *Journal of Spinal Disorders & Techniques*.

[21] Chang D. G., Kim J. H., Ha K. Y., Lee J. S., Suk J. S., Suk S. I. (2015). Posterior hemivertebra resection and short segment fusion with pedicle screw fixation for congenital scoliosis in children younger than 10 years: greater than 7-year follow-up. *Spine*.

[22] Bas C. E., Preminger J., Olgun Z. D., Demirkiran G., Yazici P., Yazici M. (2015). Safety and efficacy of apical resection following growth-friendly instrumentation in myelomeningocele patients with G: growing rod versus luque trolley. *Journal of Pediatric Orthopaedics*.

[23] Jeszenszky D., Haschtmann D., Kleinstück F. S. (2014). Posterior vertebral column resection in early onset spinal deformities. *European Spine Journal*.

[24] Xie J. M., Zhang Y., Wang Y. S. (2014). The risk factors of neurologic deficits of one-stage posterior vertebral column resection for patients with severe and rigid spinal deformities. *European Spine Journal*.

